# Periodontitis contributes to COPD progression via affecting ferroptosis

**DOI:** 10.1186/s12903-023-03397-x

**Published:** 2023-09-14

**Authors:** Kaixin Xiong, Peng Yang, Wei Wei, Jia Li, Yujia Cui, Yan Li, Boyu Tang

**Affiliations:** 1 State Key Laboratory of Oral Diseases & National Center for Stomatology & National Clinical Center for Oral Diseases, West China Hospital of Stomatology, Sichuan University, Chengdu, 610041 China; 2 Department of Cardiovascular Surgery, West China Hospital, Sichuan University, Chengdu, Sichuan P.R. China; 3 Beijing Stomatological Hospital, Capital Medical University, Beijing, China; 4 State Key Laboratory of Oral Diseases & National Center for Stomatology & National Clinical Center for Oral Diseases, Department of Conservation Dentistry and Endodontics, West China Hospital of Stomatology, Sichuan University, Chengdu, 610041 China

**Keywords:** Periodontitis, COPD, Ferroptosis, MDA, *P. gingivalis*

## Abstract

**Background:**

Periodontitis has emerged as a potential risk factor for chronic obstructive pulmonary disease (COPD). However, the precise mechanism through which periodontitis influences the progression of COPD requires further investigation. Ferroptosis is one of the crucial pathogenesis of COPD and recent researches suggested that periodontitis was associated with ferroptosis. Nonetheless, the relationship among periodontitis, COPD and ferroptosis remains unclear. This study aimed to elucidate whether periodontitis contributes to COPD exacerbation and to assess the potential impact of ferroptosis on periodontitis affecting COPD.

**Methods:**

The severity of COPD was assessed using Hematoxylin and eosin (H&E) staining and lung function tests. Iron assays, malondialdehyde (MDA) measurement and RT-qPCR were used to investigate the potential involvement of ferroptosis in the impact of periodontitis on COPD. Co-cultures of periodontitis associated pathogen *Phophyromonas gingivalis* (*P. gingivalis*) and lung tissue cells were used to evaluate the effect of *P. gingivalis* on inducing the ferroptosis of lung tissue via RT-qPCR analysis. Clinical Bronchoalveolar Lavage Fluid (BALF) samples from COPD patients were collected to further validate the role of ferroptosis in periodontal pathogen-associated COPD.

**Results:**

Periodontitis aggravated the COPD progression and the promotion was prolonged over time. For the first time, we demonstrated that periodontitis promoted the ferroptosis-associated iron accumulation, MDA contents and gene expressions in the COPD lung with a time-dependent manner. Moreover, periodontitis-associated pathogen *P. gingivalis* could promote the ferroptosis-associated gene expression in single lung tissue cell suspensions. Clinical BALF sample detection further indicated that ferroptosis played essential roles in the periodontal pathogen-associated COPD.

**Conclusion:**

Periodontitis could contribute to the exacerbation of COPD through up-regulating the ferroptosis in the lung tissue.

**Supplementary Information:**

The online version contains supplementary material available at 10.1186/s12903-023-03397-x.

## Background

Periodontitis is a chronic inflammation of periodontal supporting tissue caused by dental plaque, calculus and other local factors [1]. This condition has been confirmed to be associated with several systemic diseases, including cancers, diabetes and COPD, etc. [2, 3]. COPD is a prevalent pulmonary disease characterized by persistent airflow restriction, which can progress to pulmonary hypertension and respiratory failure [4, 5]. It is associated with an enhanced chronic inflammatory response of the airway and lung to toxic particles or harmful gases, and has a high disability rate and fatality rate [4, 6]. The incidence rate of COPD has reached 10.3% globally [7] and 13.7% in China [8].

Periodontitis has been identified as a potential risk factor for COPD. Dozens of clinical researches and meta-analysis demonstrated the positive correlation between periodontitis and COPD [3, 9–12]. Patients afflicted with periodontitis face an elevated risk of developing COPD, and there exists a positive correlation between the severity of periodontitis and the severity of COPD [11]. Periodontitis associated indices were negatively correlated with lung function indices, suggesting that poor periodontal health was related to COPD severity [13, 14]. Furthermore, risk for COPD mortality was significantly increased with the increase of periodontitis severity [10]. Nevertheless, the precise underlying mechanisms that establish the link between periodontitis and COPD remain to be fully elucidated.

Ferroptosis is a form of regulated cell death characterized by iron-dependent lipid peroxidation [15]. Excess iron especially excess ferrous iron (Fe^2+^) caused by various reasons, greatly accelerated the lipid peroxidation of polyunsaturated fatty acids [16]. The accumulation of lipid peroxides can ultimately lead to the cell membrane collapse and cell death. Research into the mechanisms governing ferroptosis is still ongoing, and recent studies have proposed that ferroptosis may contribute to the pathogenesis of COPD. It’s reported that cigarette smoke-induced ferroptosis in epithelial cells played a pivotal role in COPD pathogenesis [17]. Cigarette smoke triggers NCOA4-mediated ferroptosis in Bronchial Epithelial Cells, subsequently promoting macrophage M2 polarization and advancing COPD progression [18]. Similarly, hydrogen sulfide alleviated particulate matter-induced COPD through suppressing ferroptosis by regulating Nrf2-PPAR-ferritinophagy signaling pathway [19]. Overall, while research on the role of ferroptosis in COPD is still in its early stages, mounting evidence suggests its significant involvement in the disease’s pathogenesis.

Although research on the relationship between ferroptosis and periodontitis is limited, some evidence suggested that ferroptosis might contribute to the pathogenesis of periodontitis. *P. gingivalis*-LPS induced the ferroptosis of Human gingival fibroblasts and promoted the ferroptosis in the gingival tissues [20]. Additionally, inhibition of ferroptosis could alleviate periodontitis-induced tissue damage and bone loss [21]. These findings emphasized an association between periodontitis and ferroptosis, suggesting that periodontitis potentially exacerbated local tissue ferroptosis.

The mechanisms between periodontitis and COPD remain unclear. While positive correlations have been established between periodontitis and both COPD and ferroptosis, it is yet unexplored whether periodontitis could impact COPD progression through ferroptosis. In this study, we aimed to preliminarily explore the role of ferroptosis in the interaction between periodontitis and COPD and to provide a new therapeutic target for the controlling of periodontitis-associated COPD.

## Methods and materials

### Ethical approval

We confirm that all methods involving animals were performed in accordance with the relevant guideline and regulations. The animal study was approved by the animal research committee of West China School of Stomatology, Sichuan University (Approval number: WCHSIRB-D-2020-127). This study conformed to the Animal Research: Reporting In Vivo Experiments (ARRIVE) guidelines for animal studies. The clinical samples were collected under the approval and supervision of the Medical Ethics Committee of West China Hospital of Stomatology, Sichuan University (Approval number: WCHSIRB-D-2022-473). The experiments involving clinical sample collection were conducted in accordance with the Declaration of Helsinki. The written informed consents were signed for each participant prior to the experiments.

### *P. gingivalis* culture

*P. gingivalis* W83 was grown in hemin (5 µg/mL) (Solarbio, Beijing, China) and menadione (1 µg/mL) (Solarbio, Beijing, China) containing brain heart infusion broth (BHI) (Oxiod, Basingstoke, UK) anaerobically (37 °C, 85% N2, 10% H2, 5% CO2). 10^^9^ CFU/mL bacteria were collected for following use.

For mice oral infection, the bacterial precipitates obtained after centrifugation were resuspended in a 2% carboxymethylcellulose (CMC) solution.

In the case of the bacteria-cell co-culture experiments, the bacterial precipitates obtained after centrifugation were resuspended in 10% fetal bovine serum (FBS)-containing DMEM and then co-cultured with cells at a multiplicity of infection (MOI) of 100. [22, 23].

### Experimental mouse model

Mouse models were established to investigate the promotion ability of periodontitis on COPD and to preliminarily explore the potential involvement of ferroptosis in this process. Specific pathogen free (SPF) C57BL/6J mice (Male, 7-week-old) were purchased from Dashuo Biological Technology (Chengdu, China) and were divided into several groups randomly (5 mouse/group).

Periodontitis models were constructed through tying the 5 − 0 silk ligatures around the maxillary second molars and orally infecting with *P. gingivalis* (10^^9^ CFU/mL, 0.2mL/mice) every other day. The ligature placement procedure was carried out after mice were anesthetized with 1.25% Tribromoethanol (0.2ml/10 g, Nanjing Aibei Biotechnology Co., Ltd). One week later, COPD mouse models were started to be constructed with newly purchased cigarettes (Marlboro, 12 mg tar/1.0 mg nicotine; Philip Morris, Richmond, VA) and porcine pancreatic elastase (PPE, RHAWN, R028727). Mice were anesthetized through inhalation of 2% isoflurane and underwent the intratracheal injection of phosphate-buffered saline (PBS) dissolved PPE (2.5 U/mouse each time, totally, 5U/mouse) twice. Every day, mice were exposed to mainstream cigarette smoke (CS) for 2 h through the Cigarette Smoke Generators TSE system (TSE Systems China). For the shorter-term COPD model (earlier stage of COPD), the daily CS exposure time were 2 weeks, and for the longer-term COPD model (later stage of COPD), the daily CS exposure time were extended to 4 weeks [24–27].

### Lung function test

After anesthesia (1.25% Tribromoethanol, 0.2ml/10 g, Nanjing Aibei Biotechnology Co., Ltd) and trachea cannula, mice underwent the lung function test with the EMMS eSpira Forced Maneuvers system (CRFM100, EMMS, Alton, UK) [28] according to the manufacture’s instruction. Then, mice were sacrificed by cervical dislocation under anesthesia status (1.25% Tribromoethanol, 0.2ml/10 g, Nanjing Aibei Biotechnology Co., Ltd). Data were shown as the fold change of Forced expiratory volume at the end of 0.05s (FEV0.05) and ratio of forced expiratory volume at the end of 0.05s to forced vital capacity (FEV0.05/FVC) compared to those in Control group.

### H&E staining

After fixation of 24 h, the lung tissues of mice were processed and embedded in paraffin. Subsequently, five-micrometer sections were prepared. H&E staining was performed to observe the morphological changes and to assess the severity of the disease. This was carried out following the manufacturer’s instructions using the H&E staining kit (Solarbio, G1120).

### Perls staining

Perls Staining was carried out to observe the iron accumulation in the lung tissue with the Prussian blue kit (Yeasen Biotechnology (Shanghai), 60533ES60) according to the instruction [29]. Briefly, 4 μm-thick lung tissue sections were deparaffinized for 2 h at 60 °C and subsequently washed in distilled water for approximately 1 min. The lung tissues were then incubated in the freshly prepared Perls staining solution (a mixture of equal volumes of potassium ferrocyanide and hydrochloric acid solutions) for 20–40 min, followed by rinsing with distilled water for 2–5 min. Subsequently, the nuclei of the lung tissues were stained using Nuclear Fast Red solution for 1–5 min and washed with distilled water for 1–5 s. The samples were examined under a light microscope.

### Iron assay

The iron contents in the lung tissue homogenates were quantified by Iron assay [29]. Total Iron and Ferrous Iron contents were detected with Total Iron Colorimetric Assay Kit (Elabscience, E-BC-K772-M) and Ferrous Iron Colorimetric Assay Kit (Elabscience, E-BC-K773-M) according to the manufactures’ instructions. The measurements were carried out by recording the optical density (OD) value at a wavelength of 593 nm.

### Malondialdehyde (MDA) detection

MDA served as a measurement marker of oxidative stress, particularly, it acts as one of the main biomarkers for lipid peroxidation assessment [30, 31]. MDA detection was carried out to analyze the lipid peroxidation phenomenon of the ferroptosis procedure in the periodontitis-affected COPD model with the MDA kit (Solarbio, BC0025).

### RT-qPCR

RT-qPCR analysis was conducted to assess the expression levels of ferroptosis-associated genes. Total RNA extraction and reverse transcription were carried out with RNA extraction kit **(**Yeasen, 19221ES50) and RNA reverse kit (Yeasen Biotechnology (Shanghai), 11141ES60), respectively. Subsequently, the cDNA samples underwent RT-qPCR cycling with the RT-qPCR kit (Syber Green, Yeasen Biotechnology (Shanghai), 11201ES08) according to the kit’s instruction. The gene expression levels were calculated with 2^−∆∆CT^ method [32]. The specific primers for the tested genes were designed using PrimerBanK(https://pga.mgh.harvard.edu/primerbank/) and listed in Supplementary Table S1.

### In vitro coculture experiment

Fresh single lung tissue cell suspensions from mice with periodontitis induced by silk ligatures or healthy control mice were co-cultured with *P. gingivalis* in 10% FBS-containing DMEM medium for 24 h. The effects of the periodontal pathogen *P. gingivalis* on the ferroptosis of these single lung tissue cell suspensions were subsequently analyzed using the RT-qPCR method as described earlier.

### Clinical BALF sample analysis

Clinical BALF samples were collected from COPD patients at the Clinical Microbiology Lab of West China Hospital. Totally, 53 clinical BALF samples were collected. The characterization of the clinical subjects in this study were concluded in Supplementary Table S2. After centrifugation, the precipitations were used for DNA extraction and RNA extraction, the supernatants were collected for Iron contents and MAD levels confirmation. The obtained gDNA were subjected to RT-qPCR with the *P. gingivalis* specific primers [33] (Forward: 5’-AGGCAGCTTGCCATACTGCG-3’; Reverse: 5’-ACTGTTAGCAACTACCGATGT-3’) for analyzing whether the COPD BALF samples were with periodontitis-associated state (If the CT value was more than 35, it meant that no *P. gingivalis* was detected in this sample and it was divided into the COPD-no P. g group; If the CT value was less than 35, it meant that *P. gingivalis* existed in this sample and it was divided into the COPD-P. g group). The obtained RNAs were used for gene expression levels analysis with the specific primers, which were designed using PrimerBanK(https://pga.mgh.harvard.edu/primerbank/) and listed in Supplementary Table S3.

### Statistical analysis

Statistical analyses were conducted using various methods depending on the nature of the data. For the evaluation of differences between two groups in animal models and clinical samples, the t-test or Kruskal-Wallis analysis was employed after a homogeneity test of variance using Levene’s test. For the multiple-group animal models and four-group in *vitro P. gingivalis*-cell co-culture experiments, differences analysis among multiple groups were carried out with one-way ANOVA and post hoc Tukey’s multiple comparisons, and for the analysis of two independent groups, t test was used. Statistical analysis was performed with a significance level of 0.05 using with GraphPad Prism7 software (version 7.00, Inc, La Jolla, USA), and then all figures were also generated with this software. There are at least three independent samples in each group and data in this research were represented as the mean ± standard deviation (SD).

## Results

### Periodontitis promoted COPD progression

**Fig. 1:**
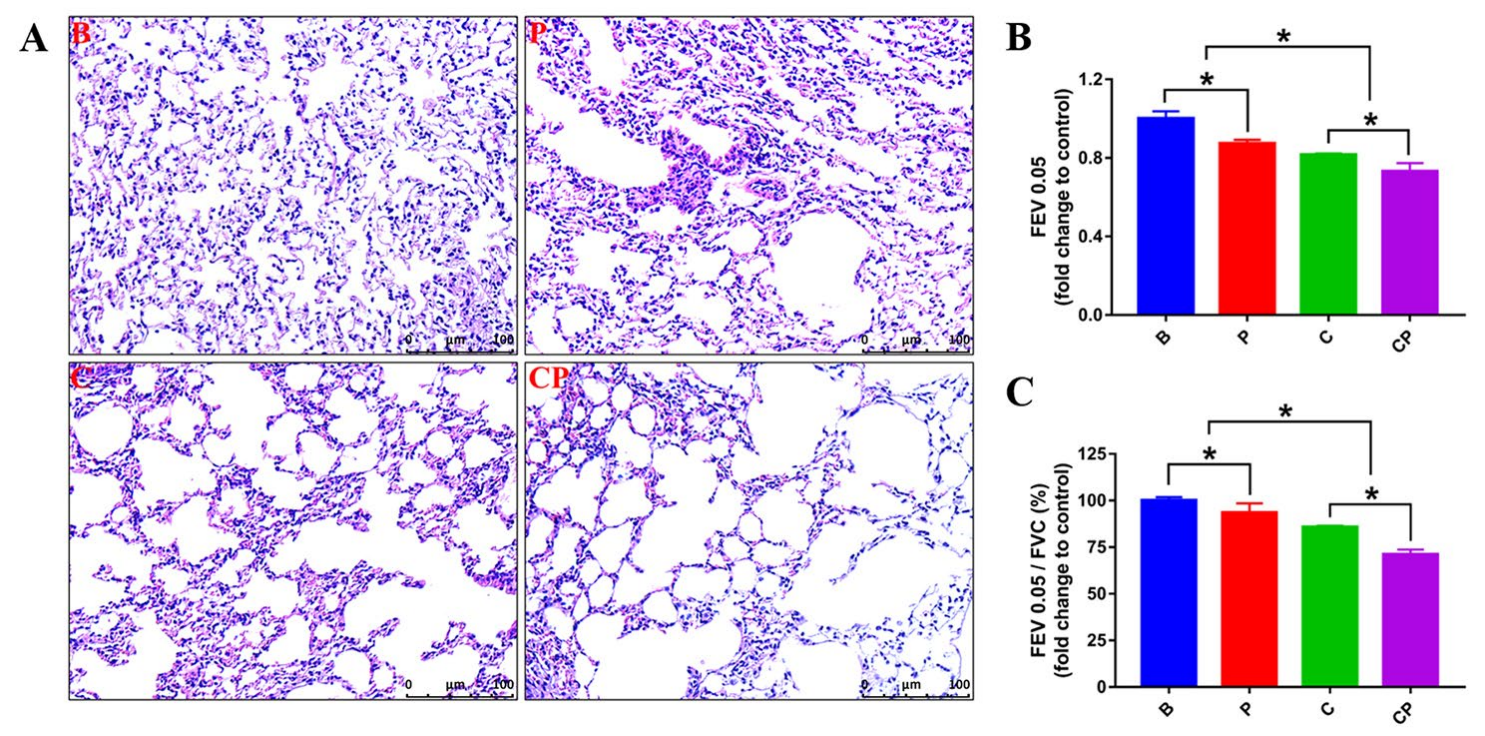
Periodontitis promoted COPD progression. Results of the earlier-stage of COPD (two weeks of daily CS exposure). (**A**): In the earlier-stage of COPD, lung lesions were observed and representative H&E images were shown. (**B**, **C**): The lung functions were compared in each group and the result were presented with the fold change of FEV0.05 value and FEV0.05/FVC value to control group. *: *p* < 0.05. B: Blank Control, P: periodontitis, C: COPD, CP: COPD with periodontitis

To assess whether periodontitis contributes to the progression of COPD, we established mouse models categorized into four groups: Blank Control (B Group), Periodontitis (P Group), COPD (C Group), and COPD with periodontitis (CP Group). Initially, we examined the periodontal status and confirmed the successful establishment of periodontitis by assessing tooth looseness. Furthermore, H&E observation of the jaw further confirmed the successful construction of periodontitis. As shown in Supplementary Figure S1, both P group and CP group showed significant alveolar bone destruction. Notably, the CP group displayed a little more serious alveolar bone destruction compared to the P group, suggesting that the presence of COPD can exacerbate periodontitis to some extent (Supplementary Figure S1). Subsequently, we evaluated the impact of periodontitis on COPD in the earlier stage of COPD (2 weeks of daily CS exposure). As shown in Fig. 1, periodontitis notably accelerated the progression of COPD. H&E observation suggested that the CP group exhibited the most pronounced dilated and fractured alveolar walls (Fig. 1A), indicating the worsening of COPD under the coexisting condition of periodontitis. Lung function test results revealed that the CP group had decreased FEV0.05 value and FEV0.05/FVC value compared to the C group, indicating that periodontitis aggravated the lung function decrease of COPD (Fig. 1B, C).

### Ferroptosis happened in the procedure of periodontitis promoting COPD

**Fig. 2:**
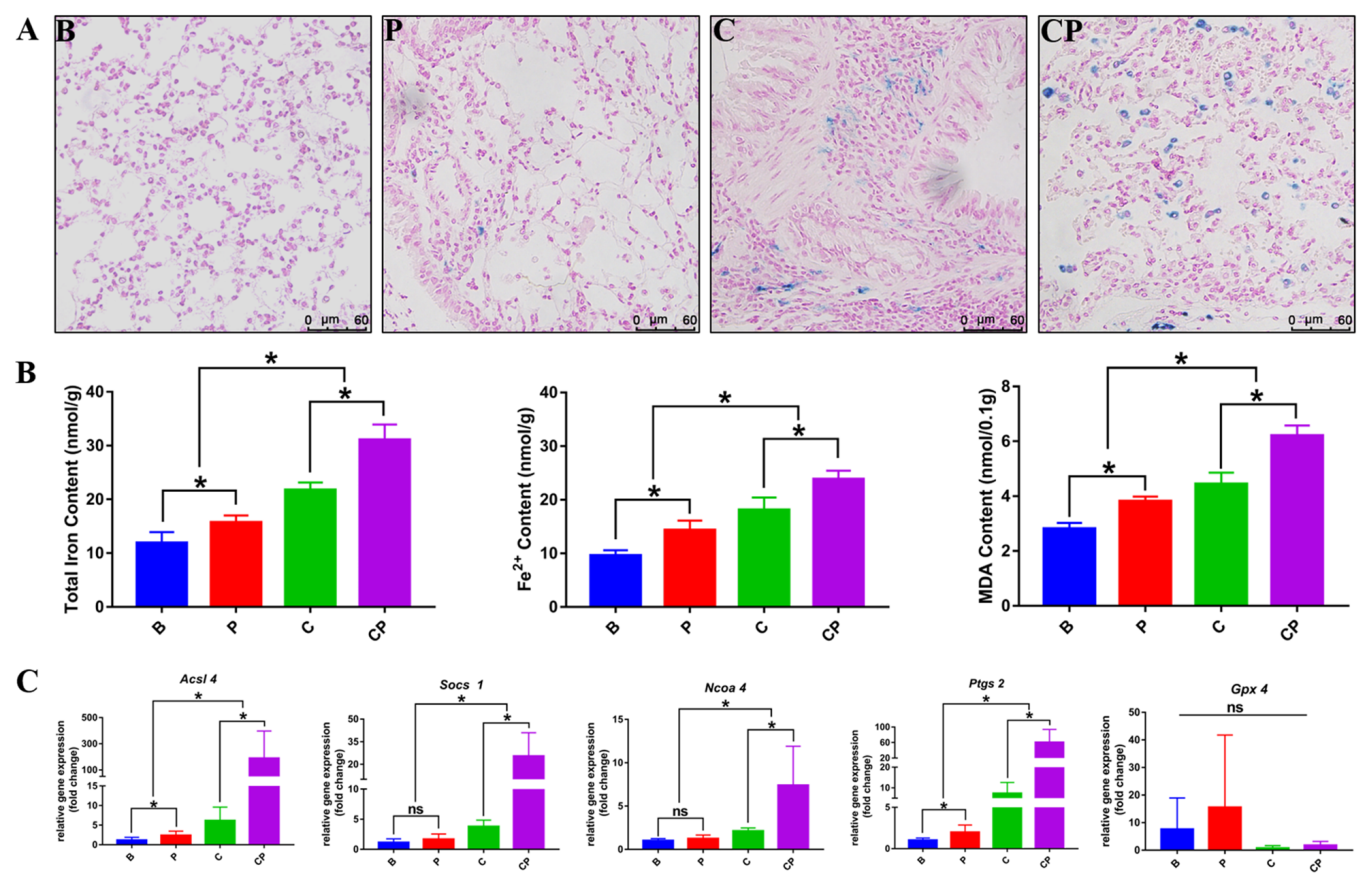
Periodontitis exacerbated COPD through promoting ferroptosis in the lung. Results of the earlier-stage of COPD (two weeks of daily CS exposure). (**A**): In the earlier-stage of COPD, the accumulation of iron in the lung were observed through Perls staining and the representative images were shown. (**B**): The total iron content, ferrous iron content and lipid peroxides content (MDA content) in the lungs were calculated to compare the ferroptosis in each group quantitatively. (**C**): The relative mRNA expression of ferroptosis related genes in the lung tissues were analyzed by RT-qPCR, including *Acsl4*, *Gpx4*, *Socs1*, *Ncoa4* and *Ptgs2*. *: *p* < 0.05, ns: not significant. B: Blank Control, P: periodontitis, C: COPD, CP: COPD with periodontitis

To preliminarily ascertain the role of ferroptosis in periodontitis-affected COPD, we carried out the ferroptosis associated detections and focused on the Iron contents, lipid peroxidation and ferroptosis-related gene expressions in the earlier-stage of COPD. Notably, periodontitis promoted the ferroptosis in the lungs of COPD mice (Fig. 2). As shown in the Fig. 2A, Perls staining demonstrated apparent iron accumulation in the lung of COPD mice (C and CP groups) and periodontitis further promoted the increase of iron accumulation. Iron contents measurements suggested that periodontitis significantly elevated both the total iron and ferrous iron contents in the lungs of COPD mice quantitatively (Fig. 2B). Moreover, we assessed oxidative stress and lipid peroxidation status by quantifying the levels of MDA, a lipid peroxidation product implicated in triggering ferroptosis. As shown in Fig. 2B, COPD with periodontitis showed significantly increased MDA contents compared to COPD mice, suggesting that periodontitis promoted the oxidative stress in the ferroptosis procedure. Further analysis of ferroptosis-associated gene expressions using RT-qPCR consistently demonstrated that periodontitis increased the expression of ferroptosis-promoting genes, including *Acsl4*, *Socs1*, *Ncoa4* and *Ptgs2*, while exhibiting not significant impact on the expression of the ferroptosis-inhibiting gene *Gpx4* (Fig. 2C). Collectively, these findings indicated that periodontitis could contribute to the progression of COPD by increasing ferroptosis in the lung to a certain extent.

### The promoting effect of ferroptosis on periodontitis affecting COPD was prolonged over time

**Fig. 3:**
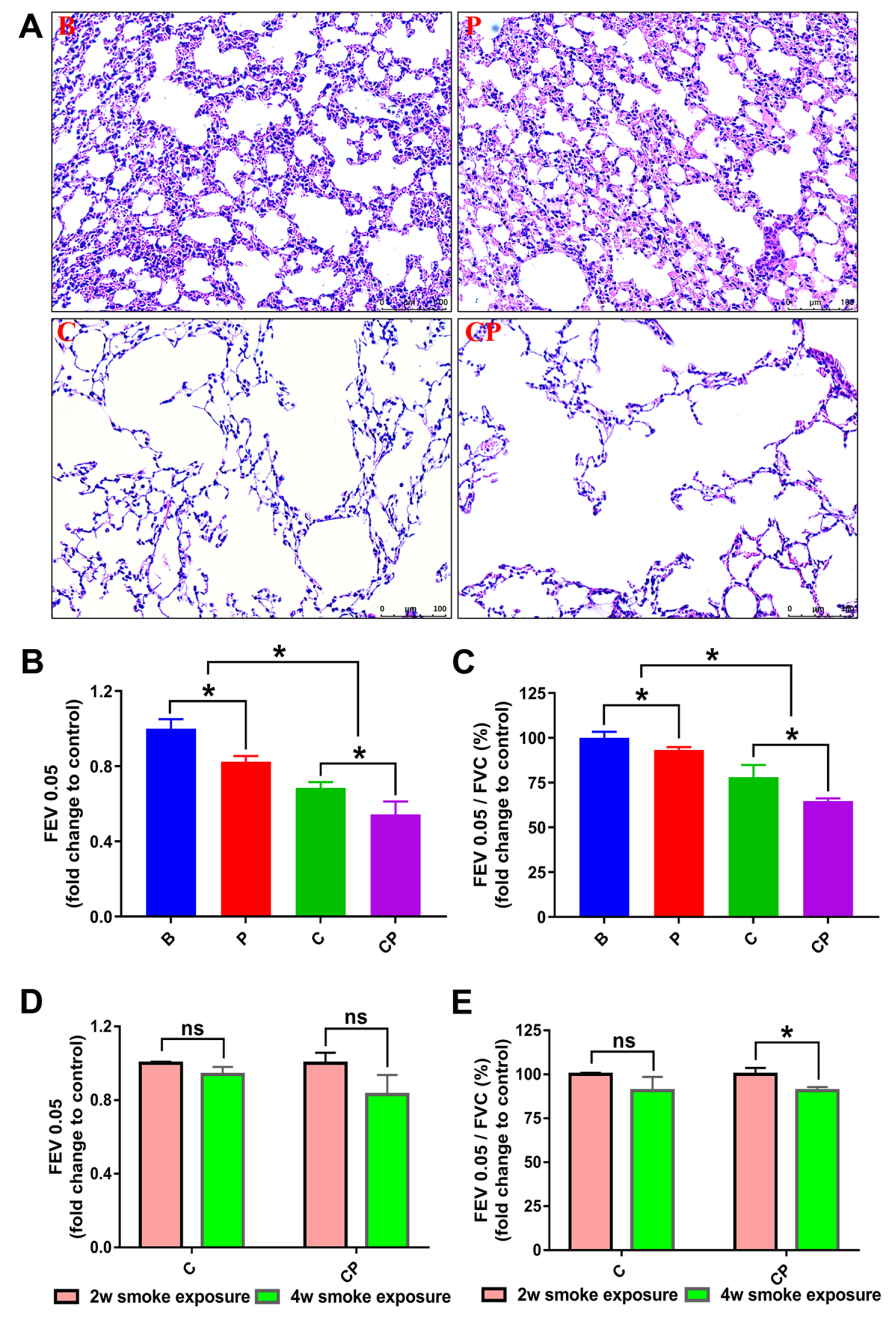
The promotion effect of periodontitis on COPD was prolonged over time. Following, we further extended the daily smoke exposure time to 4 weeks to analyze whether the promotion effect of periodontitis on COPD was prolonged over time. (**A**): In the later-stage of COPD, H&E staining observation demonstrated more apparent COPD lesion in the lung, and periodontitis showed more serious promotion effect on the lesion. (**B**, **C**): The lung functions were compared among the four groups in the later-stage of COPD, and the result were presented with the fold change of FEV0.05 value and FEV0.05/FVC value to control group. (**D**, **E**): The lung function test results of COPD group and COPD with periodontitis group in the later-stage of COPD were further compared with those in corresponding earlier-stage COPD. *: *p* < 0.05, ns: not significant. B: Blank Control, P: periodontitis, C: COPD, CP: COPD with periodontitis

To investigate whether the exacerbating effect of periodontitis on COPD progression persisted over time, we compared the COPD severity between COPD group and COPD with periodontitis group in the later-stage of COPD (4 weeks of daily CS exposure). As illustrated in Fig. 3, in the later-stage of COPD, with the increase of CS exposure time, the reinforcing effect of periodontitis on COPD was more pronounced. Lung tissue lesions exhibited further aggravation in the CP group (Fig. 3A). Moreover, periodontitis further exacerbated the lung function decrease in the CP group, resulting with further decreased FEV0.05 value and FEV0.05/FVC compared to the earlier-stage of COPD (Fig. 3B-E).

**Fig. 4:**
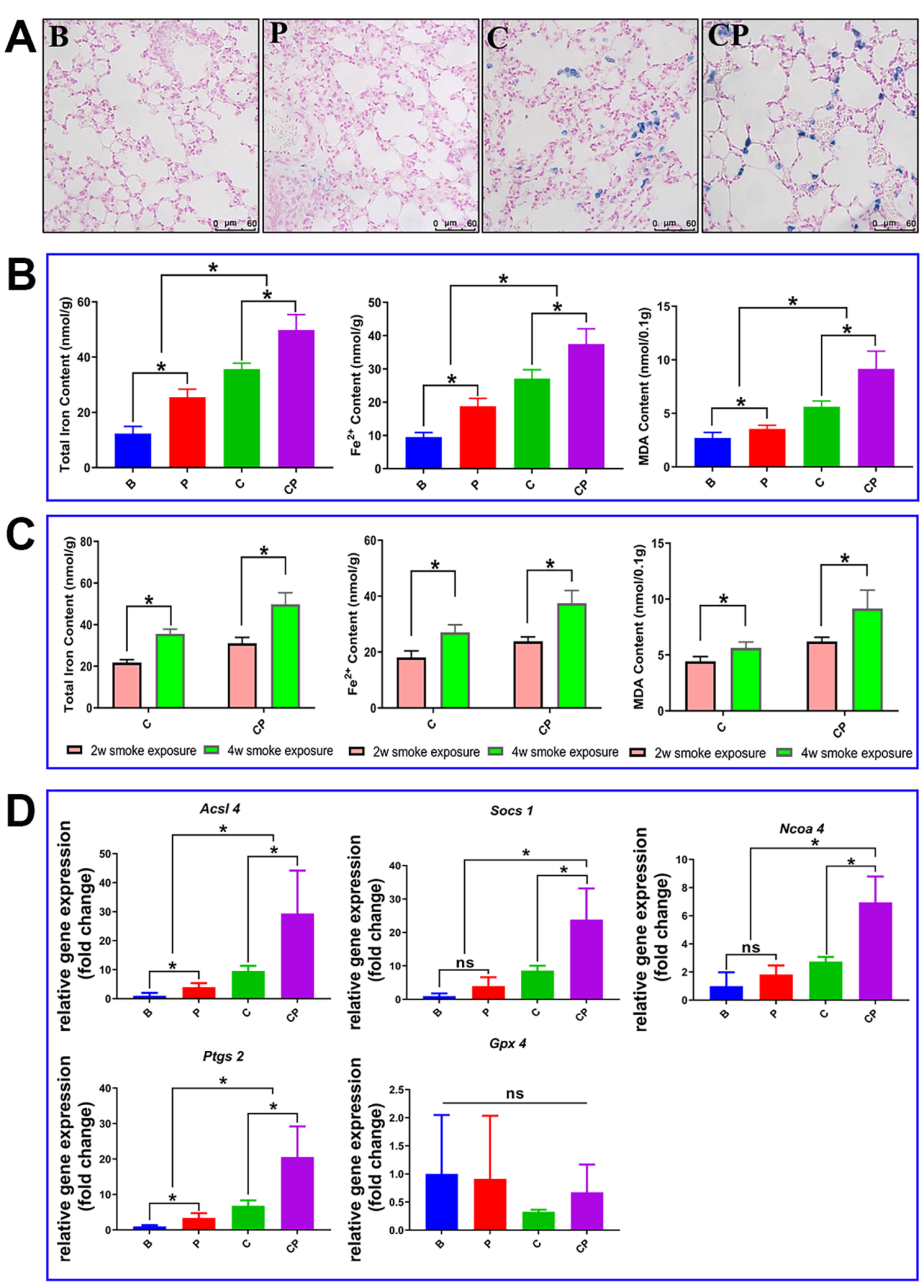
The promotion effect of periodontitis on the ferroptosis in the lung was prolonged over time. Results of the later-stage of COPD (four weeks of daily CS exposure). (**A**): In the later-stage of COPD, the iron accumulation was increased in the lung and the representative Perls staining images were shown. (**B**): In the later-stage of COPD, the total iron content, ferrous iron content and MDA content in the lungs were shown quantitatively. (**C**): In the later-stage of COPD, the total iron content, ferrous iron content and MDA content in the lungs of COPD group and COPD with periodontitis group were further quantitatively compared with their corresponding earlier-stage groups. (**D**): In the later-stage of COPD, the relative mRNA expression of ferroptosis-related genes including *Acsl4*, *Gpx4*, *Socs1*, *Ncoa4* and *Ptgs2* were compared by RT-qPCR analysis. *: *p* < 0.05, ns: not significant. B: Blank Control, P: periodontitis, C: COPD, CP: COPD with periodontitis

We also evaluated the ferroptosis phenomenon in the later-stage of COPD. Remarkably, the promotion effect of periodontitis on ferroptosis of COPD lung was also prolonged over time. There were more evident iron accumulations in the lungs of both C group and CP group, and periodontitis apparently further increased the iron accumulation (Fig. 4A). Total iron and ferrous iron analysis also suggested that in the later-stage of COPD, both total iron and ferrous iron contents were significantly increased in the lungs compared to the earlier-stage of COPD, and periodontitis could further significantly increase the total iron and ferrous iron contents (Fig. 4B, C). MDA detection revealed that periodontitis elevated the MDA contents in the lungs of COPD and this elevating effect increased with the time (Fig. 4B, C). Gene expression analysis in the longer-term COPD models demonstrated that periodontitis upregulated the expression of ferroptosis-promoting genes, providing further confirmation that periodontitis exacerbates COPD by promoting ferroptosis (Fig. 4D). These findings collectively suggested that periodontitis could intensify COPD by upregulating ferroptosis, including promoting iron accumulation, oxidative stress and lipid peroxidation production, and ferroptosis-associated genes’ expressions in the lung. Importantly, this exacerbating effect became more prominent over time.

### Periodontitis associated pathogen *P. gingivalis* could activate ferroptosis in the lung single cell suspensions

**Fig. 5:**
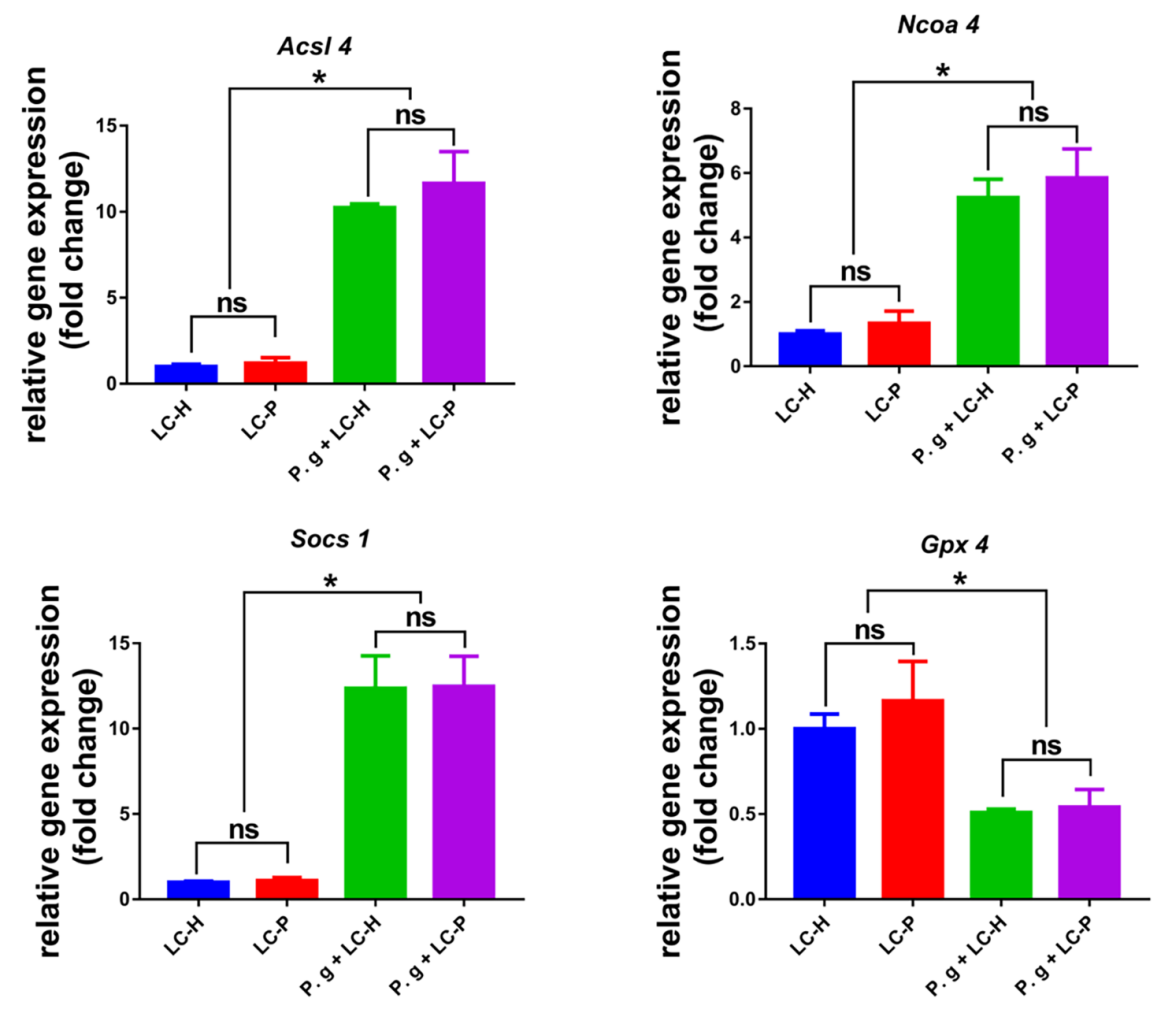
Periodontitis associated pathogen *P. gingivalis* could activate ferroptosis related genes in the lung single cell suspensions. Periodontitis associated pathogen *P. gingivalis* were cocultured with the lung single cell suspensions from health mice or ligature-induced periodontitis mice, and the expression levels of ferroptosis-related genes were analyzed by RT-qPCR. *: *p* < 0.05, ns: not significant. LC-H: Lung single cells from health mice, LC-P: Lung single cells from ligature-induced periodontitis mice

To investigate whether periodontitis associated pathogen *P. gingivalis* had the potential to activate the ferroptosis of lung, we further cocultured *P. gingivalis* and lung single cell suspensions for 24 h in vitro and then we evaluated the effects of *P. gingivalis* on the expression levels of ferroptosis-associated genes. As shown in Fig. 5, upon exposure to *P. gingivalis* stimulation, the expressions of ferroptosis-promoting genes *Acsl4*, *Ncoa4* and *Socs1* were significantly up-regulated and ferroptosis-inhibiting gene *Gpx4* was significantly down-regulated in lung single cell suspensions obtained from both healthy mice and ligature-induced periodontitis mice. While, there were not significant difference in the gene expressions between lung single cell suspensions from the healthy mice and ligature-induced periodontitis mice, regardless of the presence or absence of *P. gingivalis* stimulation. These findings emphasized the pivotal role of the periodontitis-associated pathogen *P. gingivalis*, but not ligature itself, in inducing the ferroptosis of lung.

### Enhanced iron and MDA levels and expressions of ferroptosis-related genes in clinical periodontal pathogen-containing COPD BALF samples

**Fig. 6:**
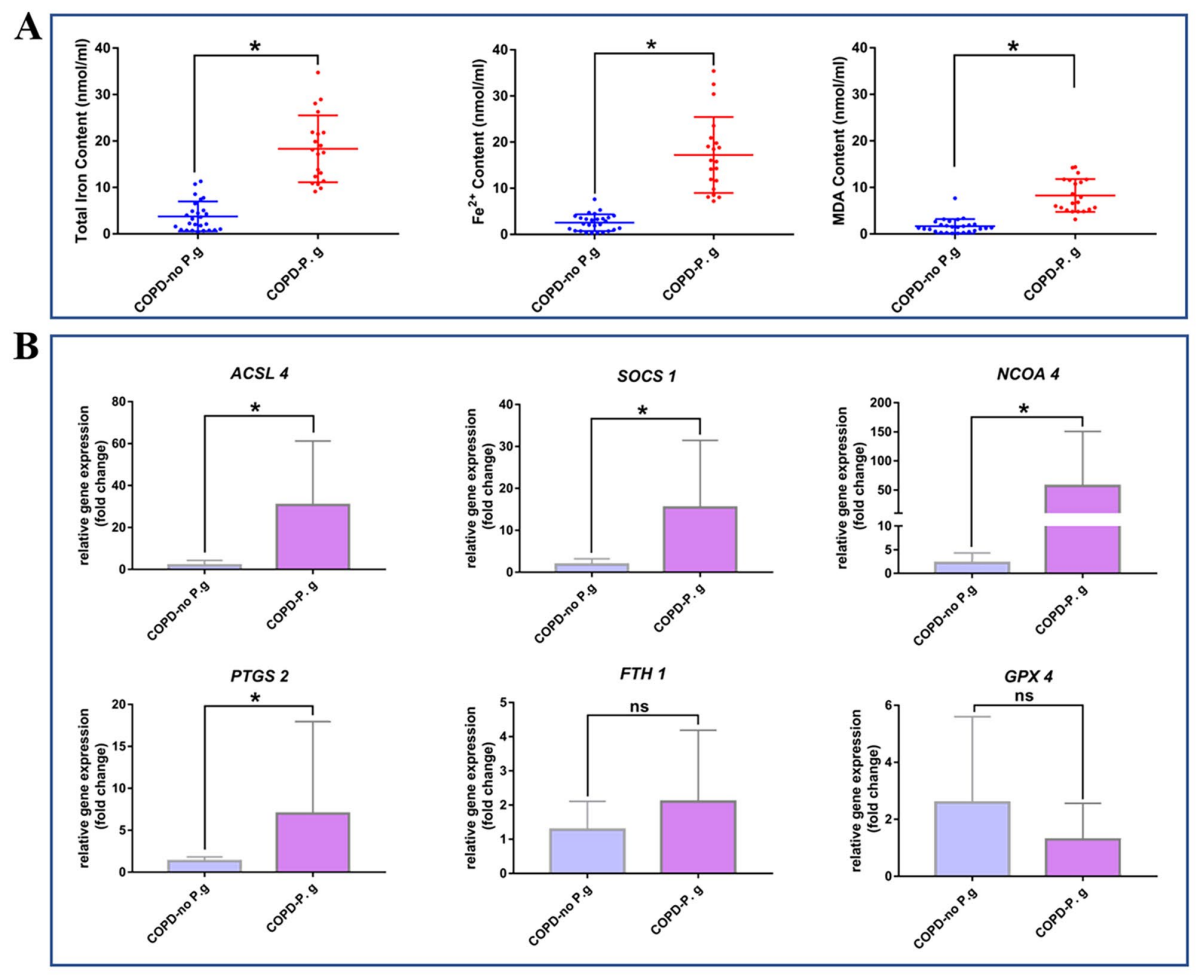
Enhanced Iron and MDA levels and ferroptosis-related genes expressions in clinical COPD with periodontal pathogen BALF samples. (**A**): The total iron content, ferrous iron content and lipid peroxides content (MDA content) in the BALF samples were quantitatively calculated. (**B**): The relative mRNA expression of ferroptosis related genes in the BALF samples were compared by RT-qPCR analysis. *: *p* < 0.05, ns: not significant

These aforementioned data collectively suggested that periodontitis exacerbated the progression of COPD through promoting ferroptosis. To further confirm the pivotal roles of ferroptosis in COPD patients with clinical evidence of periodontal pathogen presence, we conducted assessments of iron contents, MDA levels and ferroptosis-associated gene expressions in clinical BALF samples. In total, we collected 53 BALF samples from individuals with COPD. Upon *P. gingivalis* detection analysis, 25 samples exhibited the presence of *P. gingivalis* and were categorized as the COPD-P. g group, while the remaining 28 samples showed the abscence of *P. gingivalis* and were divided into the COPD-no P.g group (control group). As shown in Fig. 6A, the supernatants of the COPD-P. g group exhibited notably elevated levels of total iron, ferrous iron, and MDA. These findings suggested that the presence of the periodontal pathogen *P. gingivalis* corresponds with a heightened degree of ferroptosis in COPD. In line with these observations, the gene expression results also found that the expression levels of ferroptosis-promoting genes, including *ACSL4*, *PTGS2*, *SOCS1* and *NCOA4* were up-regulated in periodontal pathogen-existence COPD BALF samples (Fig. 6B). Collectively, our findings provided compelling evidence that in the human disease environment, ferroptosis might indeed play a crucial role in COPD with periodontitis.

## Discussion

Periodontitis and COPD are two common chronic inflammatory diseases that have been shown to have a significant association. Individuals afflicted with periodontitis are at a heightened susceptibility for developing COPD [11]. Furthermore, periodontitis treatment can lead to improvements in lung function and reduce the severity of COPD symptoms [34–36]. These findings suggest that managing periodontitis may have a beneficial impact on the overall health of individuals with COPD. However, the mechanism by which periodontitis affects COPD progression has not been fully understood.

In current research, we observed the effect of periodontitis on COPD progression through constructing animal models. Consistent with the clinical conclusions, our experimental results also confirmed that periodontitis could promote COPD progression in the animal model. Under the condition of COPD with periodontitis, mice showed increased expansion and destruction of pulmonary alveoli and decreased lung function data (Fig. 1) compared to COPD without periodontitis. Moreover, the promoting effect of periodontitis on COPD was increased with time. With the prolonged duration of the disease, the destruction of pulmonary alveoli was further aggravated, and the pulmonary function was further reduced (Fig. 3). Meanwhile, the promoting effect of periodontitis on COPD was further increased (Fig. 3). Therefore, it could be concluded that periodontitis exacerbated COPD with a time-dependent manner. However, the promoting mechanism is unclear till now.

Ferroptosis is a type of regulated cell death that is characterized by the accumulation of iron-dependent lipid peroxides, culminating in oxidative damage and cell death [15]. Ferroptosis is believed to play a role in a variety of diseases [17, 37–39]. In recent years, researchers have focused on the role of ferroptosis on COPD pathogenesis. In COPD, there is an increase of oxidative stress due to the chronic inflammation and lung tissue damage caused by exposure to cigarette smoke or other harmful particles [40]. This oxidative stress can result with lipid peroxidation and the production of MDA [41]. Lipid peroxides was the representative characteristic of ferroptosis and MDA acts as one of the main biomarkers for evaluating oxidative stress and lipid peroxidation [30, 31]. Consequently, iron accumulation and MDA levels can serve as representative markers indicating the occurrence of ferroptosis. Previous investigations have underscored the pivotal role of ferroptosis in COPD pathogenesis. Iron accumulation and enhanced lipid peroxidation were observed in the COPD mouse model and in Human Bronchial Epithelial cells, signifying ferroptosis’ crucial involvement in driving COPD pathogenesis [17]. Moreover, increased MDA levels in COPD have been confirmed to be associated with disease severity and lung function impairment [42]. In CS extract stimulated Human Bronchial Epithelial cells (BEAS-2B) and lung tissues of CS induced COPD rats, iron and MDA were significantly increased, suggesting that ferroptosis was involved in lung epithelial cell damage and COPD pathogenesis [43]. Furthermore, inhibition of ferroptosis with ferroptosis inhibitor ferrostatin-1 indicated decreased injury in the CS extract stimulated BEAS-2B cells [43]. In short, accumulating evidence strongly supports the crucial role of ferroptosis in the intricate pathogenesis of COPD.

Currently, there are relatively limited reports addressing the connection between periodontitis and ferroptosis. Nonetheless, a few studies have provided insights into the potential promotion of ferroptosis by periodontitis. For instance, investigations have indicated that *P. gingivalis*-LPS could induce the ferroptosis in the human gingival fibroblasts and gingival tissue [20]. Moreover, the expression of ferroptosis-related genes, including *NCOA4*, *SLC1A5*, *HSPB1*, etc. were significantly upregulated in the human periodontitis-gingival samples compared to the periodontal healthy normal gingival samples [44]. Additionally, periodontitis-level butyrate could lead to ferroptosis in periodontal ligament fibroblasts via activating *NCOA4*-mediated ferritinophagy and destroying the iron homeostasis, leading to further development of periodontitis [45]. In short, these data indicated that periodontitis could promote the occurrence of ferroptosis phenomenon.

At present, there are no studies indicated that ferroptosis acted as the mechanism mediating periodontitis to promote COPD progression, however, previous studies have proved that ferroptosis served as the important mechanism mediating the impact of periodontitis on systemic diseases. Through bioinformatics analysis and experimental validation, Pan et al. found that in the pathological mechanism and treatment of periodontitis with type 2 diabetes mellitus, ferroptosis acted as a crucial target [46]. In Non-alcoholic fatty liver disease (NAFLD), *P. gingivalis* could induce ferroptosis in hepatocytes and further worsen liver lesions, and targeting at ferroptosis could acted as a new strategy for clinical treatment and prevention of NAFLD with periodontal inflammatory state [47, 48]. Drawing from these findings, we speculated that periodontitis might also contribute to COPD progression by promoting ferroptosis in the lung. In this study, we found that periodontitis could promote the accumulation of iron and the production of lipid peroxide MDA in COPD lung tissues, and up-regulate the expression of ferroptosis-related genes (Fig. 2). Notably, ferroptosis was more severe in the lung of COPD with periodontitis. Impressively, this exacerbating effect of periodontitis on ferroptosis in COPD lungs displayed an escalating trend over time (Fig. 4). These results preliminarily suggested ferroptosis played a pivotal role in the periodontitis-driven progression of COPD. Furthermore, in our in vitro experiments, we found that the co-culture of periodontal pathogens with lung tissue single-cell suspension could affect the expression of ferroptosis-related genes in lung cells. Specifically, expressions of genes associated with ferroptosis promotion (*Acsl4*, *Ncoa4*, and *Socs1*) were up-regulated, while the expression of gene associated with ferroptosis inhibition (*Gpx4*) was downregulated (Fig. 5). Importantly, these shifts in ferroptosis-related gene expressions in lung cells were only occurred in the presence of the pathogenic bacteria *P. gingivalis*. In the absence of pathogenic microorganisms, the gene expression profile of lung cells in the periodontitis mouse model induced solely by silk ligature did not significantly alter (Fig. 5). This experiment underscored the important role of periodontitis, particularly periodontal pathogenic bacteria, in driving ferroptosis in the lung, and consequently, exacerbating the progression of COPD. Subsequently, we further verified our conclusion in BALF samples of clinical COPD patients. Notably, the presence of periodontal pathogenic bacteria was associated with significantly elevated iron and MDA levels in COPD BALF samples, alongside a notable upregulation of ferroptosis-promoting genes’ expressions (Fig. 6). Collectively, those above data demonstrated that, to some degree, periodontitis could exacerbate the progression of COPD through promoting the ferroptosis in the lung.

### Limitations

The current study offers preliminary evidence indicating that periodontitis may contribute to the exacerbation of COPD by promoting ferroptosis in the lung. While, the further intricate molecular mechanism among periodontitis, ferroptosis and COPD is still in our further exploration. And we’ll further demonstrate the detailed molecular mechanisms among them by blocking the ferroptosis-associated genes in vitro and in vivo in our future experiments.

### Clinical applications and scope for future scope

In this study, our data suggested that periodontitis could promote COPD exacerbation through up-regulating the ferroptosis in the lung tissue. Therefore, in clinical treatment, targeting at oral health management and ferroptosis might be a new practical strategy for COPD prevention or control, especially for those with periodontal inflammation. And in the future, further investigations could explore the effects of some ferroptosis-inhibiting agents on periodontitis-associated COPD in vitro and in vivo. These endeavors have the potential to shed more light on the intricate interplay among periodontitis, ferroptosis and COPD, ultimately contributing to more effective therapeutic approaches for the diseases.

## Conclusion

In summary, we observed that periodontitis could aggravate COPD progression in the mouse models and identified that ferroptosis played a crucial role in mediating periodontitis promoting COPD for the first time. The periodontitis state, especially the periodontitis pathogenic bacteria aggravated COPD severity through increasing the ferroptosis in the lung. Therefore, targeting at oral health management and ferroptosis inhibition might be a new practical strategy for COPD prevention or control, especially for those with periodontal inflammation.

### Electronic supplementary material

Below is the link to the electronic supplementary material.

Supplementary Material 1

## Data Availability

All data sets generated and/or analyzed in the current study are available from the corresponding author on reasonable request.

## Abbreviations

COPD: Chronic Obstructive Pulmonary Diseases
MDA: malondialdehyde
*P. gingivalis*: *Phophyromonas gingivalis*
BALF: Bronchoalveolar Lavage Fluid
CS: Cigarette Smoke
