# Supplementary material for: Periodontitis contributes to COPD progression via affecting ferroptosis
Source: BMC Oral Health. 2023 Sep 14;23:664. doi: 10.1186/s12903-023-03397-x (PMC10500905; doi:10.1186/s12903-023-03397-x)
Supplement: Supplementary file 1 — Supplementary Material 1 [file 12903_2023_3397_MOESM1_ESM.docx]

**Periodontitis contributes to COPD progression via affecting ferroptosis**

**Authors:** Kaixin Xiong^1,#^, Peng Yang^2,#^, Wei Wei^1^, Jia Li^1^, Yujia Cui^1^, Yan Li^1,^*, Boyu Tang^1,3,^*

1 State Key Laboratory of Oral Diseases & National Center for Stomatology & National Clinical Center for Oral Diseases, West China Hospital of Stomatology, Sichuan University, Chengdu, 610041, China

2 Department of Cardiovascular Surgery, West China Hospital, Sichuan University, Chengdu, Sichuan, P.R. China.

3 State Key Laboratory of Oral Diseases & National Center for Stomatology & National Clinical Center for Oral Diseases & Department of Conservation Dentistry and Endodontics, West China Hospital of Stomatology, Sichuan University, Chengdu, 610041, China

^#^ Kaixin Xiong and Peng Yang contribute equally to this work.

***Corresponding Author:**

Dr. Yan Li, State Key Laboratory of Oral Diseases & National Center for Stomatology & National Clinical Center for Oral Diseases, West China Hospital of Stomatology, Sichuan University, Chengdu, 610041, China (Email: [feifeiliyan@163.com](mailto:feifeiliyan@163.com)). ORCID ID: 0000-0002-2323-8105

Dr. Boyu Tang, State Key Laboratory of Oral Diseases & National Center for Stomatology & National Clinical Center for Oral Diseases & Department of Conservation Dentistry and Endodontics, West China Hospital of Stomatology, Sichuan University, Chengdu, 610041, China (Email: [boyutang@126.com](mailto:boyutang@126.com)). ORCID ID: 0000-0003-0022-5181


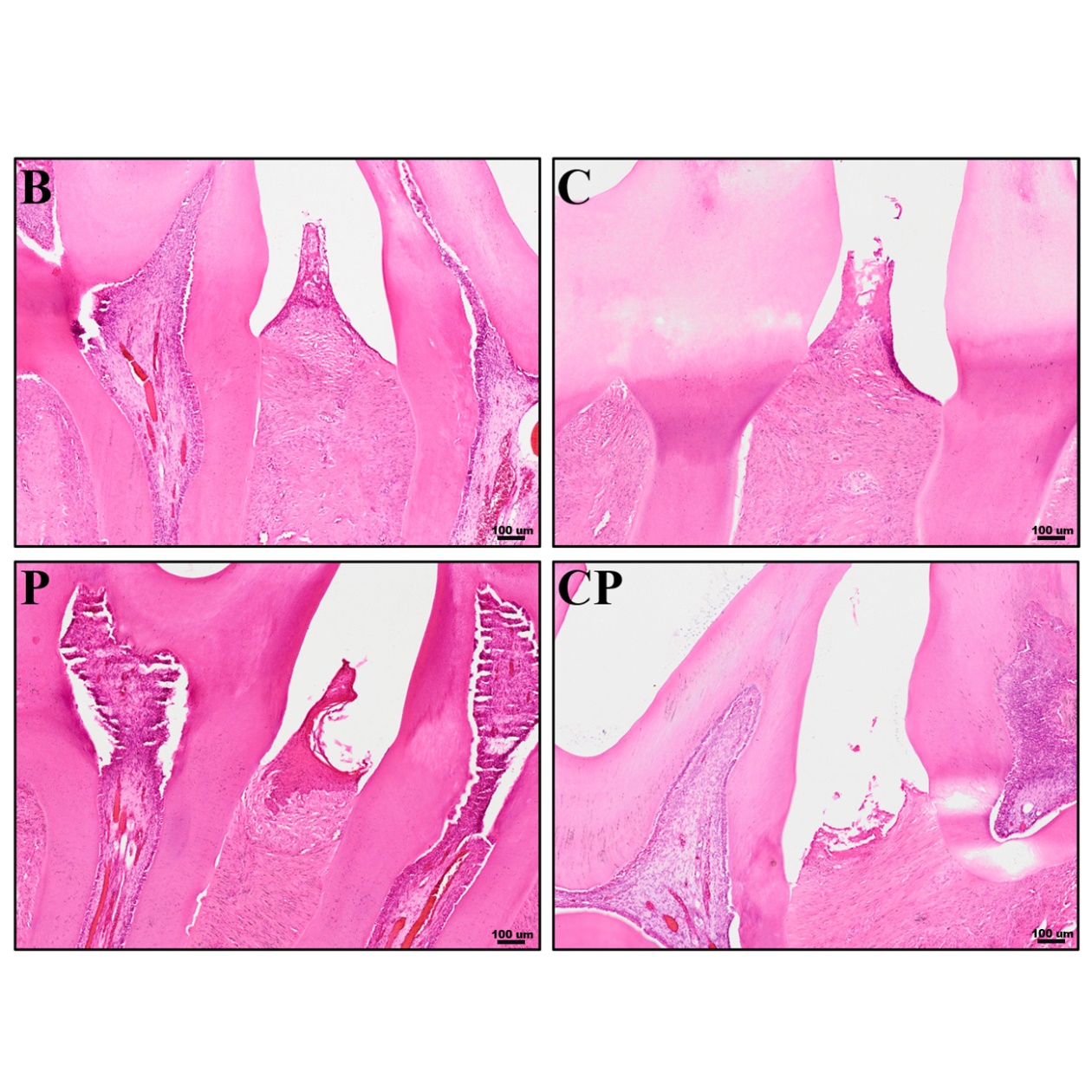


**Supplementary Figure S1: COPD could also slightly contribute to the severity of periodontitis.** Periodontal pathological changes were observed and representative H&E images were shown. B group showed completely healthy alveolar bone and C group showed very slightly absorbed alveolar bone. Both P and CP groups demonstrated evident alveolar bone destruction, and compared to P group, the alveolar bone destruction was a little more serious in CP group. B: Blank Control, P: periodontitis, C: COPD, CP: COPD with periodontitis.

**Supplementary Table S1: RT-qPCR primers of ferroptosis-related genes used for mice samples in this study**

| Genes | Primers | Sequences (5’–3’) |
| --- | --- | --- |
| *Gapdh* | *Gapdh*-F  *Gapdh*-R | AGGTTGTCTCCTGCGACTTCA  CCAGGAAATGAGCTTGACAAA |
| *Acsl4* | *Acsl4*-F  *Acsl4*-R | CCTGAGGGGCTTGAAATTCAC  GTTGGTCTACTTGGAGGAACG |
| *Socs1* | *Socs1*-F  *Socs1*-R | CTGCGGCTTCTATTGGGGAC  AAAAGGCAGTCGAAGGTCTCG |
| *Ncoa4* | *Ncoa4*-F  *Ncoa4*-R | GCCCTACAATGTGAGTGATTGG  ACTGGTGCAAGGCTCGTTG |
| *Ptgs2* | *Ptgs2* -F  *Ptgs2* -R | TGAGCAACTATTCCAAACCAGC  GCACGTAGTCTTCGATCACTATC |
| *Gpx4* | *Gpx4*-F  *Gpx4*-R | GCCTGGATAAGTACAGGGGTT  CATGCAGATCGACTAGCTGAG |

**Supplementary Table S2: Characterization of the clinical subjects in this study**

|  | COPD-no P.g group | COPD-P.g group |
| --- | --- | --- |
| Number of samples | 28 | 25 |
| Age (year) | 70.18±10.28 | 69.92±11.9 |
| Sex | 2 females and 26 males | 1 female and 24males |
| Hypertension | 4 samples | 5 samples |
| Diabetes | 3 samples | 3 samples |
| Heart disease | 5 samples | 7 samples |
| Infectious diseases | None | None |
| Lung Tumors | None | None |

**Supplementary Table S3: RT-qPCR primers of ferroptosis-related genes used for in human samples in this study**

| Genes | Primers | Sequences (5’–3’) |
| --- | --- | --- |
| *GAPDH* | *GAPDH*-F  *GAPDH*-R | ACAACTTTGGTATCGTGGAAGG  GCCATCACGCCACAGTTTC |
| *ACSL4* | *ACSL4*-F  *ACSL4*-R | CATCCCTGGAGCAGATACTCT  TCACTTAGGATTTCCCTGGTCC |
| *SOCS1* | *SOCS1*-F  *SOCS1*-R | TTTTCGCCCTTAGCGTGAAGA  GAGGCAGTCGAAGCTCTCG |
| *NCOA4* | *NCOA4*-F  *NCOA4*-R | ACAGTTGCATAAGCCGTCACC  TGAGCCTGCTGTTGAAGTGTC |
| *PTGS2* | *PTGS2* -F  *PTGS2* -R | CTGGCGCTCAGCCATACAG  CGCACTTATACTGGTCAAATCCC |
| *FTH1* | *FTH1*-F  *FTH1*-R | CCCCCATTTGTGTGACTTCAT  GCCCGAGGCTTAGCTTTCATT |
| *GPX4* | *GPX4*-F  *GPX4*-R | GAGGCAAGACCGAAGTAAACTAC  CCGAACTGGTTACACGGGAA |
